# Oxidants Induce a Corticosteroid-Insensitive Phosphorylation of Histone 3 at Serine 10 in Monocytes

**DOI:** 10.1371/journal.pone.0124961

**Published:** 2015-04-23

**Authors:** John A. Marwick, Corina Tudor, Nadia Khorasani, Charalambos Michaeloudes, Pankaj K. Bhavsar, Kian F. Chung

**Affiliations:** 1 Section of Airways Disease, National Heart & Lung Institute, Imperial College, London, United Kingdom; 2 MRC Centre for Inflammation Research, Queens Medical Research Institute, University of Edinburgh Medical School, Edinburgh, United Kingdom; 3 NIHR Biomedical Research Unit, Royal Brompton Hospital, London, United Kingdom; Lund University, SWEDEN

## Abstract

Oxidative stress enhances inflammation and reduces the effectiveness of corticosteroids, but the inflammatory signalling pathways induced by oxidants remain ill-defined. Phosphorylation of histone 3 at serine 10 (H3-Pser10) marks out a subset of inflammatory genes for transcription, several of which are induced in oxidant-associated inflammation. However, the influence of oxidants or of corticosteroids on this modification remains unknown. We assessed the regulation of H3-Pser10 by oxidants and lipopolysaccharide (LPS) in human blood monocytes and lung macrophages and the effectiveness of its abolition in controlling inflammatory gene expression in cells from asthmatic subjects compared to corticosteroids alone. Both oxidants and LPS promoted the induction of H3-Pser10 which was unaffected by corticosteroids. The induction of H3-Pser10 was mediated through p38α mitogen-activated protein kinase (MAPK) and IκB kinase 2 (IKK-2) signalling. Consequently, inhibitors of p38α MAPK or IKK-2 used in combination with dexamethasone were more effective at controlling inflammatory gene expression from monocytes and lung macrophages from asthmatic patients than the corticosteroid alone. Therefore, reduction of H3-Pser10 by inhibition of p38α MAPK or of IKK-2 may provide greater anti-inflammatory control than corticosteroids alone in oxidant-associated inflammation such as severe asthma.

## Introduction

The failure of corticosteroids to control the persistent lung inflammation in severe asthma and chronic obstructive pulmonary disease (COPD) is widely attributed to oxidative stress [1–3]. Therefore, a detailed understanding of how oxidants impact on inflammatory signalling is needed.

Histone modifications, such as acetylation, control the recruitment and access of transcriptional complexes to gene promoters [4] and are a major node of inflammatory control. Oxidants heighten inflammatory responses, in part, by inactivating histone deacetylase 2 (HDAC2) [5]. This prevents histones at inflammatory gene promoters from being de-acetylated and the inflammatory genes silenced. Oxidants also activate stress pathways including kinases such as p38 mitogen-activated protein kinase (MAPK) [6,7], phosphatidylinositol 3-kinase (PI3K) [8,9] and transcription factors such as NF-κB [10,11]. This, combined with elevated histone acetylation, culminates in uncontrolled inflammatory transcription which ‘locks’ the cell into a persistent inflammatory state.

However, histone acetylation is not the sole histone modification that regulates transcriptional control. Phosphorylation of histone 3 at serine 10 (H3-Pser10) is also important in controlling inflammatory gene transcription [12]. This serves to recruit NF-κB to the gene promoter of a subset of immediate-early pro-inflammatory genes (such as IL-6, CXCL-8 and CCL-2) and enables subsequent acetylation at lysine residues 9 and 14 [13]. Several pathways regulate H3-Pser10, including oxidant-sensitive pathways such as the p38 MAPK and IκB Kinase (IKK) pathways [13,14]. Corticosteroids may also influence H3-Pser10 through the induction of dual specificity MAPK phosphatases (DUSP-1 or MKP-1) which reduces p38 MAPK activation [15]. Consequently, as oxidants activate p38 MAPK signalling and impair corticosteroid function, H3-Pser10 may be involved in the reduced control and chronicity of oxidant-associated inflammation. However, the impact of oxidants and corticosteroids on the regulation of H3-Pser10 remains unknown.

In this study, we use monocytes from healthy volunteers to examine the impact of oxidative stress and corticosteroids on the induction of H3-Pser10. Thereafter, in order to study the effect of oxidant-associated inflammation which is not fully controlled by corticosteroids, we chose to examine monocytes and lung macrophages from patients with asthma, particularly severe asthma. The monocytes and macrophages from these patients are less sensitive to the anti-inflammatory effects of corticosteroids [16,17] which is concordant with poor therapeutic responsiveness of asthma control by corticosteroid treatment in these patients [18] and there is evidence of increased oxidative stress [19]. Our aim was to examine whether a reduction in the induction of H3-Pser10 in the cells from the asthmatic subjects produced a greater control of inflammatory cytokine expression than a corticosteroid alone.

Our findings indicate that oxidants induce H3-Pser10 which was not inhibited by dexamethasone and reducing H3-Pser10 using the selective p38α MAPK inhibitor, SB239063, and the IKK-2 inhibitor, TPCA-1, is more effective at controlling the expression of inflammatory mediators in cells from asthmatic patients than corticosteroids alone.

## Materials and Methods

### Subjects

Healthy volunteers had no history of respiratory disease had normal spirometric results. Patients with severe asthma were prospectively recruited from the Severe Asthma clinic at the Royal Brompton Hospital, London. Patients with severe asthma needed either continuous or near-continuous oral corticosteroids, high-dose inhaled corticosteroids, or both to achieve a level of mild-to-moderate persistent asthma, and by 2 or more minor criteria (Table 1) [20]. Patients with non-severe asthma had controlled asthma while using up to 2,000 μg/day or equivalent of inhaled beclomethasone. Current smokers and ex-smokers of greater than 5 pack-years of smoking were excluded. Asthmatic subjects underwent fiberoptic bronchoscopy during which bronchoalveolar lavage was performed and lung macrophages were obtained. All the subjects were free from upper respiratory tract infections and acute exacerbations within 3 months before bronchoscopy. All patients provided written informed consent to participate in this study, which was approved by the Brompton, Harefield and NHLI Research Ethics Committee (08/H0708/29).

**Table 1:** Characteristics of non-severe and severe asthmatic subjects.

	Non-severe asthmatics (n = 11)	Severe asthmatics (n = 10)
**Sex (M:F)**	**6:5**	**2:8**
**Age (years)**	**38.8 ± 13.4**	**48.2 ± 13.6**
**Duration of Asthma (years)**	**29.2 ± 15.3**	**27.3 ± 10.7**
**FEV**_**1**_ **(% predicted)**	**84.6 ± 11.9**	**74.3 ± 26.4**
**Bronchodilator response (% increase in FEV**_**1**_ **)**	**14.8 ± 7.6**	**25.2 ± 21.1**
**Log PC**_**20**_ **methacholine (mg/ml)**	**1.2 ± 1.9**	**1.1 ± 0.5**
**Prednisolone dose (mg/day)**	**none**	**11.1 ± 12.9** ^**a**^
**Beclomethasone Dipropionate equivalent (μg/day)**	**677.7 ± 463.1**	**1362.5 ± 483.8**

^a^6 severe asthmatic subjects were on oral prednisolone at the time of the study. Data shown as mean ± SEM.

### Isolation of peripheral blood monocytes and alveolar macrophages

Peripheral blood mononuclear cells (PBMCs) were isolated from the peripheral blood of normal healthy and asthmatic subjects using discontinuous Percoll gradients. The monocytes were purified (>95%) using the Monocyte Isolation kit II (Miltenyi Biotech). Alveolar macrophages (AMs) were isolated from the bronchoalveolar lavage (BAL) from non-severe asthmatic subjects and severe asthmatic subjects (Table 1) by selective adhesion yielding macrophage purity of >97%. Cells were cultured in phenol red free RPMI 1640 with 1% autologus serum, 5% L-glutamine and 5% penicillin/streptomycin (Gibco).

### Reagents

The kinase inhibitors SB239063 (a p38α MAPK inhibitor), TPCA-1(an IKK-2 inhibitor) were purchased from Merck Biosciences. Dexamethasone, hydrogen peroxide (H_2_O_2_) and pyocyanin were purchased from Sigma and 1,2,3,4-Oxatriazolium, 5-amino-3-(3,4-dichlorophenyl)- chloride (GEA 3162) was purchased from Calbiochem. The anti-Histone H3 P-Ser10 and anti-Histone H3 (Millipore) antibodies were purchased from Millipore and the anti-phospho-p38 MAPK, anti-p38 MAPK anti-phospho-IKK1/2 and anti-IKK-2 were purchased from Cell Signaling Technology.

### Cell treatment

Monocytes and AMs were cultured overnight with inhibitors added 1 hour prior to stimulation. The cells were then stimulated with LPS (Sigma), or oxidants as detailed in the figures. The expression of selected proteins was knocked down by siRNAs (Applied Biosystems) using lipofectamine 2000 (Invitrogen). The siRNAs against p38α MAPK, MSK-1, MSK-2, IKK-1 and IKK-2 were introduced to the cells as per the manufacturer’s instructions and the effective knockdown of protein levels was observed 72h after transfection as assessed by Western blotting for the respective target protein levels.

### Western Blotting

Cytosolic extracts were isolated using a hypotonic extraction buffer; 50mM Tris pH 8.0, 2mM EDTA pH 8, 0.1% NP40, 10% Glycerol, protease inhibitors (Merck Biosciences) and phosphatase inhibitors (Roche Applied Sciences). Nuclear extracts were isolated using a 1% SDS lysis buffer; 50mM Tris pH 8.0, 5mM EDTA pH 8, 1% SDS, protease inhibitors (Merck) and phosphatase inhibitors (Roche). Samples were run on 4–12% Bis-Tris gels (Invitrogen) and transferred onto Optitran BA-S 83 nitrocellulose (Whatman; GE Heathcare Life Sciences). Blots were developed using ECL (GE Healthcare Life Sciences).

### Chromatin Immunoprecipitation (ChIP) Assay

ChIP assays were performed on 1x10^6^ cells using EZ-Magna ChIP kits (Millipore) as per the manufacturer’s instructions. Sonication was performed with a MSE Soniprep 150 (MSE UK Ltd, London, UK) using 9 x 10 second bursts at 10 amplitude microns with 60 seconds on ice between pulses. Immunoprecipitation of H3-Pser10 was performed overnight with the ChIPAb+ anti-H3-Pser10 antibody (Millipore). qRT-PCR was performed using SYBR Green Fastmix (Applied Biosystems) with primers designed around NF-κB promoters upstream of the CXCL-8, IL-6, TNFα and CCL-2 genes (synthesised by Eurofins). CXCL-8: 5’-GGGCCATCAGTTGCAAATC-3’ and 5’-TTCCTTCCGGTGGTTTCTTC-3’. IL-6; 5’-GCCCAGCTATGAACTCCTTCTC-3’ and 5’-CTGGCAGTTCCAGGGCTAAG-3’. TNFα; 5’-GAAGGTGCAGGGCCCAC-3’ and 5’-CTTGGTGGAGAAACCCAT-3’. CCL-2; 5’-GCTCCAGCCAAATGCATTCT-3’ and 5’-TGAGGAGGCAGCTTTGGAAGT-3’. qRT-PCR was performed using a Applied Biosystems 7500 Fast Real-Time PCR System (Applied Biosystems).

### Real Time RT-PCR

RNA extractions were performed using RNeasy kits (Qiagen) according to the manufacturer’s instructions. RNA was quantified using a nanodrop (Thermo Scientific) and 2μg of RNA was transcribed into cDNA using; 10U AMV RTase, 40U RNAsin, 25ng/ml Hexamer primer, 1mM 4xdNTPs (Promega). qRT-PCR was performed using 5μl of cDNA with TaqMan Gene Expression master mix (Applied Biosystems) and pre-designed Taqman probes for IL-6, CXCL-8, TNFα, CCL-2 and GNB2L (Applied Biosystems) according to the manufacturer’s instructions using a Corbett Rotor-Gene Thermocycler (Corbett Research Ltd).

### Statistical analysis

Statistical analysis was performed by one-way ANOVA with Tukeys post-hoc test using GraphPad Prism 5 software (GraphPad Software Inc). Differences were considered significant at P < 0.05.

## Results

### Oxidative stress induces a sustained induction of H3-Pser10 and augments LPS induce H3-Pser10 in monocytes

**Fig 1:**
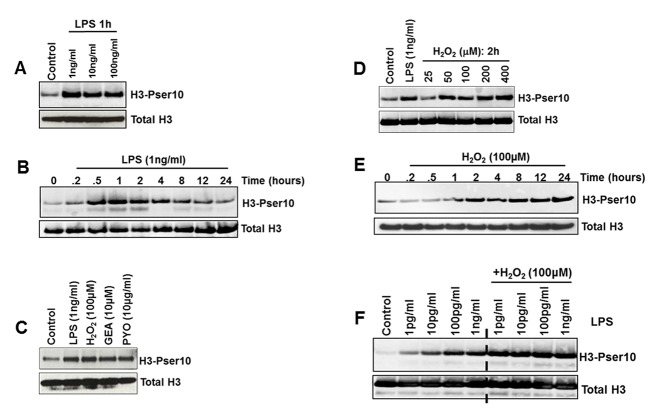
Induction of H3-Pser10 by LPS and oxidants. Panels A-F show the induction of H3-Pser10 measured by western blot analysis in blood monocytes from a normal healthy subject, with the dose-dependent effect of LPS (A), time-course (B), the comparative effect of LPS and oxidants H_2_O_2_, GEA and pyocyanin (C), dose-dependency and time-course effect of H_2_O_2_ (D, E) and the interaction of H_2_O_2_ and LPS (F).

LPS and both exogenously-derived oxidants (via H_2_O_2_ and GEA, a superoxide, nitric oxide and peroxynitrite generator) and endogenously-derived oxidants (via pyocyanin, a superoxide generator via the mitochondria) induced H3-Pser10 in human blood monocytes from healthy volunteers (Fig 1). The induction by LPS was relatively transient, with a reduction by 4–8h (Fig 1A and 1B). In contrast, the oxidant-mediated by H_2_O_2_ was more prolonged than that elicited by LPS, lasting up to 24h (Fig 1E). In addition, low concentrations of LPS (<1ng/ml) mediated a greater induction of H3-Pser10 in the presence of H_2_O_2_ (Fig 1F).

### Oxidant induction of H3-Pser10 in monocytes is mediated by p38α MAPK via MSK1 signalling and IKK-2 which is unaffected by corticosteroids

**Fig 2:**
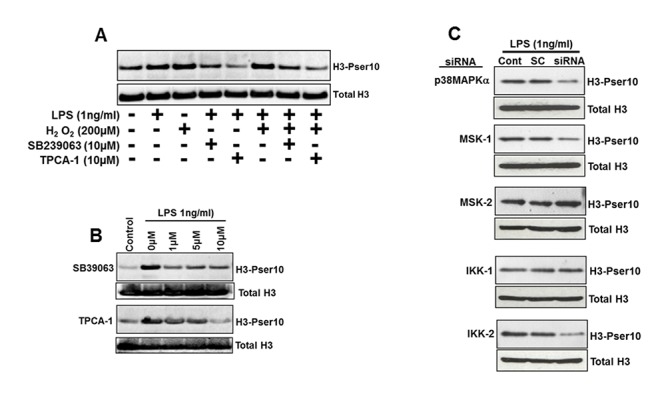
Induction of H3-Pser-10 is dependent in p38α MAPK and IKK-2 signalling. Panel A shows the expression of H3-Pser10 measured by western blot from blood monocytes from a healthy normal subject induced by H_2_O_2_ and LPS, and the effect of the p38α MAPK inhibitor SB239063 and the IKK-2 inhibitor TPCA-1. Panel B shows the concentration dependent reduction of LPS-induced H3-Pser10 expression by SB239063 and TPCA-1. Panel C shows the reduction of LPS-induced H3-Pser10 by siRNA knockdown of p38α MAPK, MSK-1 and IKK-2 protein expression but not by knockdown of MSK-2 or IKK-1 protein expression.

The induction of H3-Pser10 by both LPS and H_2_O_2_ was impaired by selective inhibition of p38 MAPKα using SB239063 and IKK-2 using TPCA-1 in human blood monocytes from healthy volunteers (Fig 2A and 2B). SB239063 is selective for the p38 α and β isoforms (IC50 = 44nM) with ~200 fold selectivity compared to other MAP Kinases and no effects on the γ and δ p38 MAPK isoforms. The α p38 MAPK isoform is highly expressed in monocytes and macrophages, whilst the protein expression of the β,γ and δ isoforms are very low to undetectable [21]. Therefore the effects of SB239063 in the monocytes are mediated through the inhibition of the p38α MAPK isoform. The IKK inhibitor TPCA-1 is relatively selective for the IKK-2 isoform (IC_50_ = 17.9nM), exhibiting a 22-fold selectivity over the IKK-1 isoform (400nM).

**Fig 3:**
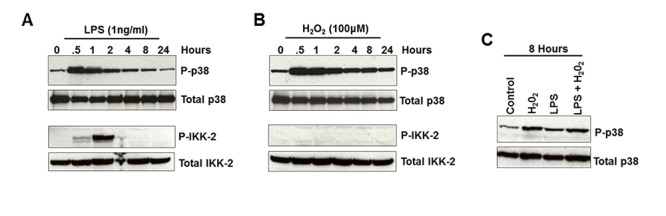
Induction of p38 MAPKα is prolonged by oxidants. Panels A and B show the phosphorylation of p38α MAPK and IKK-2 by LPS and by H_2_O_2_ respectively on Western blot of blood monocytes from a healthy subject. Panel C shows the persistence of p38α MAPK phosphorylation after 8hours after exposure to LPS, H_2_O_2_ or a combination of LPS and H_2_O_2_.

Phosphorylation of nuclear proteins via the 38α MAPK pathways may be mediated directly or through downstream signalling mediators including the mitogen- and stress-activated protein kinases 1 and 2 (MSK1/2). Selective knockdown of p38α MAPK and MSK-1 but not MSK-2 protein levels using siRNA reduced the LPS-mediated induction of H3-Pser10 confirming the involvement of p38α MAPK and MSK-1 (Fig 2C). IKK-1 may also be involved in the regulation of H3-Pser10 [14]. However, selective knock-down of IKK-2 but not IKK-1 protein reduced the LPS-mediated induction of H3-Pser10, confirming the involvement of IKK-2 in the induction of H3-Pser10 by LPS (Fig 2C). Both LPS and H_2_O_2_ induced the phosphorylation of p38α MAPK, with a more sustained elicitation from H_2_O_2_ (Fig 3A–3C). IKK-2 phosphorylation was only induced by LPS with H_2_O_2_ having no effect (Fig 3A and 3B)

**Fig 4:**
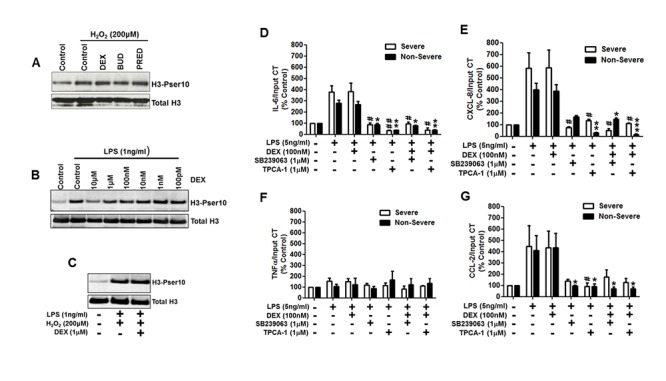
H3-Pser10 induction is associated with inflammatory gene induction and is sensitive to p38α MAPK and IKK2 inhibition but not by corticosteroid. Panels A to C show western blot analysis of H3-Pser10 expression of blood monocytes from a normal subject exposed to H_2_O_2_ (A), LPS (B) or to both H_2_O_2_ and LPs (C) in the presence of dexamethasone (DEX), budesonide (BUD) and prednisolone (PRED) Panels D-G show the mean data of the effect of SB239063, TPCA-1 and dexamethasone on the association of at the gene promoter of IL-6 (D), CXCL-8 (E), TNFα (F) and CCL-2 (G) with H3-Pser10 as assessed by ChIP of H3-Pser10 followed by qRT-PCR for the respective gene promoter in alveolar macrophages from non-severe and severe asthmatic subjects. Data is presented as the mean ± SEM. Severe asthmatics n = 5, non-severe asthmatics n = 11. * p<0.05, **p<0.01,*** p<0.001 as compared to LPS treated non-severe asthmatics; #p<0.05, ##p<0.01 as compared to LPS-treated severe asthmatics.

Corticosteroids can modulate p38α MAPK signalling through the induction of DUSP-1/MSK-1. However, dexamethasone, budesonide and prednisolone had no effect on the induction of H3-Pser10 by H_2_O_2_ in human blood monocytes from healthy volunteers (Fig 4A). Dexamethasone had no effect on the induction of H3-Pser10 mediated by LPS or by LPS in combination with H_2_O_2_ (Fig 4B and 4C), with only a supraphysiological (10μM) concentration of dexamethasone causing any reduction in H3-Pser10.

### LPS induces H3-Pser10 at the promoter region of pro-inflammatory genes which is abolished by inhibition of p38α MAPK or of IKK-2 but not by CS treatment

LPS induced H3-Pser10 at the promoter region of IL-6, CXCL-8, and CCL-2 genes in AMs from both non-severe and severe asthmatics (Fig 4D–4G). There was very little increase in the association of the TNFα gene and H3-Pser10 after LPS stimulation (Fig 4F) which is consistent with previous data suggesting that LPS-induced TNFα expression is largely H3-Pser10-independent [13]. Consistent with the failure of dexamethasone to reduce an induction of H3-Pser10 (Fig 4A–4C), dexamethasone treatment had no significant impact on the association of any of these genes with H3-Pser10. Inhibition of p38α MAPK using SB239063 significantly reduced the association of IL-6, CXCL-8 and CCL-2 with H3-Pser10 (Fig 4D, 4E and 4G). Similarly, inhibition of IKK-2 using TPCA-1 also reduced the association of IL-6, CXCL-8 and CCL-2 with H3-Pser10 (Fig 4D, 4E and 4G). The reduction in H3-Pser10 at the gene promoters by both SB23063 and TPCA-1 was comparable between non-severe and severe asthmatic AMs and there was no further effect by co-treatment with dexamethasone.

### Inhibition of p38α MAPKα or IKK-2 combined with CS suppresses the expression of pro-inflammatory mediators which are not fully suppressed by CS alone

**Fig 5:**
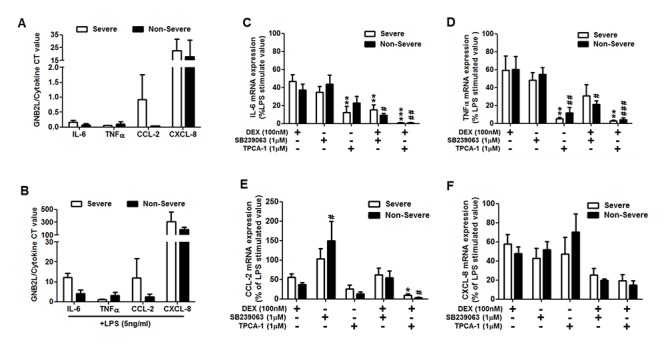
Inhibition of p38α MAPK or IKK-2 in combination with corticosteroids is more effective at reducing LPS induced inflammatory gene expression in asthmatic patients than corticosteroids alone. Panels A to B show the mean mRNA expression determined by real time qRT-PCR of IL-6, TNFα, CCL-2, and CXCL-8 in monocytes from non-severe and severe asthmatic subjects at baseline (A) or after exposure to LPS (B). Panels C to F show the mean mRNA expression of IL-6 (C), TNFα (D), CCL-2 (E), and CXCL-8 (F) in monocytes from non-severe and severe asthmatic subjects after exposure to LPS in the presence of dexamethasone (DEX), SB239063 or TPCA-1 or a combination of dexamethasone and either SB239063 or TPCA-1. Data is presented as mean ± SEM. Severe asthmatics n = 8, non-severe asthmatics n = 8. *p<0.05, **p<0.01, ***p<0.001 as compared to LPS-treated severe asthmatics; #p<0.05, ##p<0.01, ###p<0.001 as compared to LPS-treated non-severe asthmatics.

Monocytes from both non-severe and severe asthmatics expressed IL-6, TNFα, CCL-2 and CXCL-8 mRNA which was increased by exposure to LPS (Fig 5A and 5B). SB239063 elicited a comparable inhibition in the induction of IL-6, TNFα and CXCL-8 mRNA in monocytes from both non-severe and severe asthmatics as compared to dexamethasone alone (Fig 5C–5F). However, the combination of SB239063 and dexamethasone was significantly more effective at reducing the induction of IL-6 mRNA expression in both non-severe and severe asthmatics and TNFα mRNA expression in non-severe asthmatics compared to dexamethasone alone.

TPCA-1 was significantly more effective at reducing LPS-induction of IL-6 and TNFα in monocytes from non-severe and severe asthmatics compared to dexamethasone (Fig 5C and 5D). TPCA-1 co-treatment with dexamethasone was more effective at reducing the induction of IL-6 and TNFα mRNA expression compared to either treatment alone and also significantly reduced the mRNA expression of CCL-2 compared to dexamethasone treatment alone (Fig 5C–5F). Neither SB239063 nor TPCA-1 alone reduced the induction of CXCL-8 mRNA in either non-severe or severe asthmatic monocytes, even when combined with dexamethasone (Fig 5F).

## Discussion

We have shown that both oxidative stress and LPS induced H3-Pser10 in human blood monocytes through the activation of p38α MAPK which was unaffected by corticosteroids. LPS also induced H3-Pser10 through the activation of IKK-2 and inhibition of either p38α MAPK or IKK-2 was more effective at controlling inflammatory gene expression in monocytes and AMs from asthmatic subjects than a corticosteroid alone.

Although LPS and oxidative stress both induced H3-Pser10, their pattern of induction differs markedly. LPS induced a rapid and transient phosphorylation which is consistent with its role in the transcription of immediate early inflammatory genes [13] while the induction by oxidants was more prolonged, lasting up to 24 hours. This may reflect an oxidant-mediated dysregulation of the pathways that influences H3-Pser10. Indeed, oxidative stress activates a number of stress pathways that regulate H3-Pser10 including the p38α MAPK pathway [6,7], which in turn induces immediate-early (IE) gene transcription through activation of one of p38 MAPKs downstream effectors, MSK-1 [22,23]. Consistent with this, both the LPS and the oxidant induction of H3-Pser10 was abolished by selective inhibition of p38α MAPK or selective knockdown of MSK-1. The induction of H3-Pser10 by LPS also involved the activation of IKK-2 which could be blocked using the IKK-2 inhibitor TPCA-1 or selective knockdown of IKK-2 using siRNA; however, the involvement of this pathway was absent in the oxidant-mediated induction suggesting that distinct pathways regulate the induction of immediate-early genes which are stimulus dependant.

Consistent with the prolonged induction of H3-Pser10, the oxidant-mediated induction of p38 MAPK was also prolonged compared to LPS which may account, in part, for the sustained phosphorylation of H3. Oxidative stress can also reduce phosphatase activity through direct covalent modification [24]. Therefore, impairment in phosphatase activity may also directly sustain H3-Pser10 by reducing the rate of its de-phosphorylation. However the mechanism of this sustained induction remains unclear and further detailed molecular studies are needed to fully elucidate the mechanism.

Another interesting observation was the ability of oxidants also to sensitise the induction of H3-Pser10 by LPS. This suggests that the induction of H3-Pser10 by inflammatory stimuli such as bacterial products may be heightened in the presence of oxidative stress, which in turn could contribute to the heightened inflammatory response seen in oxidant associated-inflammation. Therefore, anti-inflammatory therapies that control H3-Pser10 as part for their action may be beneficial in oxidant associated-inflammation.

Corticosteroids are powerful anti-inflammatory drugs which are widely used to treat persistent inflammation in chronic diseases such as asthma. Part for their action is mediated through the expression of anti-inflammatory genes such as the phosphatase DUSP-1/MKP-1 [25] which supresses p38 MAPK signalling [15]. Consequently, part of the anti-inflammatory action of a corticosteroid may be mediated through an induction of DUSP-1/MKP-1 which in turn prevents a prolonged induction of H3-Pser10. However, corticosteroids failed to inhibit the induction of H3-Pser10 by either LPS or oxidative stress, suggesting that modulation of H3-Pser10 is not one of their major anti-inflammatory actions. Therefore, where an inflammatory microenvironment facilitates an uncontrolled production of key inflammatory mediators, driven in part by the induction of H3-Pser10, a firmer control of the inflammation may be desirable by combining corticosteroid treatment with one whose actions include a direct reduction of H3-Pser10.

Indeed, although corticosteroids are widely effective anti-inflammatory drugs they are less effective at controlling oxidant-associated chronic inflammation, such as in the lungs of severe asthmatic patients [2,17,26]. Interestingly, both p38α MAPK and IKK-2 activities are elevated in severe asthma [27,28], as are a number of H3-Pser10-regulated genes including IL-6, CCL-2 and CXCL-8 [16,17]. Therefore, direct inhibition of H3-Pser10 through modulation of p38α MAPK or IKK-2 is potentially a novel therapeutic target to improve inflammatory control in severe asthma. Indeed, selective inhibitors of p38α MAPK and IKK-2 have potent anti-inflammatory properties [21,29] and our studies also suggest that inhibition of p38α MAPK improves the reduced corticosteroid responsiveness in alveolar macrophages from severe asthmatics [30].

**Fig 6:**
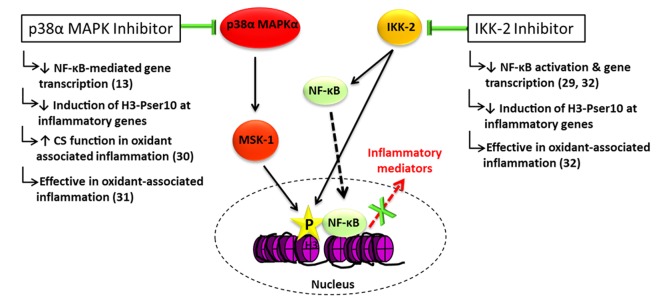
Diagram of the anti-inflammatory actions derived from the inhibition of p38α MAPK and IKK-2 through modulation of NF-κB activation and its subsequent association with inflammatory genes. Inhibition of either p38α MAPK or of IKK-2 reduces the activation of NF-κB and reduces H3-Pser10 which marks serves to flag selected immediate-early inflammatory gene for NF-κB-mediated transcription, thus reducing their expression. In addition, inhibition of p38α MAPK may increase the effectiveness of corticosteroids in asthmatic patient macrophages and both inhibitors of IKK-2 and p38α MAPK may be effective in oxidant-associated inflammation.

In this study, inhibition of p38α MAPK or of IKK-2 but not dexamethasone reduced the induction of H3-Pser10 at the gene promoters of IL-6, CCL-2 and CXCL-8 in alveolar macrophages from severe asthmatic patients. This was consistent with the failure of dexamethasone to reduce the induction of H3-ser10 mediated by LPS or oxidants. Furthermore, both inhibition of p38α MAPK and IKK-2 was more effective at reducing LPS-induced IL-6, TNFα, CCL-2 and CXCL-8 in monocytes from asthmatic subjects than corticosteroid alone, with a greater inhibition of IL-6, TNFα and CCL-2 when the inhibitors were used in combination with dexamethasone. These data support the notion that abolition of H3-Pser10 by inhibition of either p38α MAPK or IKK-2 may provide a more effective control of some inflammatory genes in asthma than corticosteroids alone. It is of course important to consider that inhibiting p38α MAPK and IKK-2 has a number of major anti-inflammatory effects, particularly in the inhibition of the activation of NF-κB (Fig 6). These will be significant factors in their functional effect in reducing inflammatory mediator expression. Inhibition of H3-Pser10 should therefore be viewed as a mechanism that both reduces the activation of NF-κB and its targeting to immediate-early inflammatory genes which are otherwise poorly controlled by corticosteroids alone patients with severe asthma.

In addition, it is critical that potential new therapies in diseases with oxidant-associated inflammation, such as in severe asthma [19], maintain their anti-inflammatory action in an oxidative microenvironment. This is evident in the case of corticosteroids whose reduction in effectiveness in severe asthma and other oxidant-associated diseases such as chronic obstructive pulmonary disease (COPD) is directly related to oxidants altering cellular and protein functions [1]. Inhibitors of both p38α MAPK and of IKK-2 have been shown to be effective in *in vivo* models of oxidative stress [31,32], however other studies suggest that IKK-2 inhibitors may not effective [33]. This serves to highlight the importance of identifying which molecules retain their effectiveness in oxidant-associated inflammation using a number of models.

In summary, we show that oxidants can promote a sustained induction of H3-Pser10 which in turn may contribute to the enhanced inflammation in oxidant-associated disease. This induction of H3-Pser10 is unaffected by corticosteroids and therefore our data also suggests that a reduction in H3-Pser10 through the inhibition of either p38α MAPK or IKK-2 in combination with corticosteroids may be significantly more effective at reducing the expression of key inflammatory mediators in oxidant-associated disease than by corticosteroids alone. Both inhibitors of p38 MAPK and IKK are in clinical development; however, detailed studies will be needed to assess if these therapies maintain their anti-inflammatory action in diseases such as severe asthma and COPD.
